# A Systematic Approach for Discovering Novel, Clinically Relevant Bacteria

**DOI:** 10.3201/eid1803.111481

**Published:** 2012-03

**Authors:** Robert Schlaberg, Keith E. Simmon, Mark A. Fisher

**Affiliations:** University of Utah School of Medicine, Salt Lake City, Utah, USA (R. Schlaberg, M.A. Fisher);; ARUP Laboratories, Salt Lake City (R. Schlaberg, K.E. Simmon, M.A. Fisher)

**Keywords:** 16S sequencing, unidentified, new species, repeated isolation, bacteria

## Abstract

Sequencing of the 16S rRNA gene (16S) is a reference method for bacterial identification. Its expanded use has led to increased recognition of novel bacterial species. In most clinical laboratories, novel species are infrequently encountered, and their pathogenic potential is often difficult to assess. We reviewed partial 16S sequences from >26,000 clinical isolates, analyzed during February 2006–June 2010, and identified 673 that have <99% sequence identity with valid reference sequences and are thus possibly novel species. Of these 673 isolates, 111 may represent novel genera (<95% identity). Isolates from 95 novel taxa were recovered from multiple patients, indicating possible clinical relevance. Most repeatedly encountered novel taxa belonged to the genera *Nocardia* (14 novel taxa, 42 isolates) and *Actinomyces* (12 novel taxa, 52 isolates). This systematic approach for recognition of novel species with potential diagnostic or therapeutic relevance provides a basis for epidemiologic surveys and improvement of sequence databases and may lead to identification of new clinical entities.

## Abstract 2

We identified 95 isolates from novel taxa that may have clinical relevance.

Broad-range PCR amplification and sequencing of the 16S rRNA gene (16S sequencing) is not only widely used as a taxonomic tool but is recognized as an effective reference method for bacterial identification. It has been used to identify novel and emerging pathogens (*1**–**4*) and to define complex microbial communities (*5**,**6*). The method has also revolutionized our understanding of microbial diversity (*7**–**9*). In clinical microbiology laboratories, 16S sequencing is useful for classifying microorganisms from pure culture (*10**,**11*). Molecular identification is especially valuable for bacteria that are slow growing, biochemically inert or variable, and fastidious, and it has also enhanced our understanding of previously unrecognized, often opportunistic pathogens (*1**,**10**,**12*).

Sequence-based identification relies on limited, yet phylogenetically informative, 16S sequence variation between related bacterial taxa. The entire 16S rRNA gene is ≈1,500 nt long (*11*); however, sequencing the 5′ third (partial 16S) generally provides sufficient taxonomic information while limiting costs (*10*). Partial 16S sequences are compared with reference libraries to determine the species with maximum similarity (*10**,**11*). The largest library is the nucleotide database hosted by the National Center for Biotechnology Information (NCBI) (*13*). Depending on their similarity to reference sequences, unknown isolates can be identified to different taxonomic levels by using interpretive guidelines published by the Clinical and Laboratory Standards Institute (CLSI) (*14*). For most taxa, sequence identity >99% with a valid reference sequence is required for species-level identification. Although this cutoff is widely used to identify isolates of the same species, a uniform cutoff for defining isolates as belonging to separate species is more controversial (*1**,**10**,**15**–**17*). Values of 99.5% to 97.0% have been proposed in the past (*12**,**15**,**17**–**22*), with more recent evidence and recommendations supporting values between 98.7% and 99.0% (*10**,**17**,**23*).

In our laboratory, as in many others, 16S sequencing is performed when morphologic and phenotypic identification is inconclusive or difficult or when it is specifically requested. By using CLSI guidelines and an NCBI nucleotide-based reference library (*24*), >90% of these isolates can be identified to the species level. However, clinical isolates belonging to as-yet-undescribed taxa are regularly encountered. Whether they represent emerging pathogens (*1*) or environmental contaminants is often difficult to determine in individual cases. Therefore, we conducted a systematic analysis of large numbers of unidentified strains to screen for novel taxa of potential clinical relevance. We reviewed partial 16S sequences from >26,000 clinical isolates to identify and characterize novel species with possible clinical significance. We identified 673 isolates that belong to as-yet-undescribed species, including 348 isolates of 95 novel taxa that were isolated from multiple patients. Repeated isolation of these undescribed organisms may indicate their clinical relevance and warrant their formal description as species.

## Methods

### Clinical Isolates

From results reported for ≈26,000 clinical isolates identified by 16S rRNA gene sequencing during February 2006–June 2010, we searched for those isolates that could not be identified to the species level by using SmartGene software (*24*) and CLSI guidelines (*14*). Phenotypic characteristics were routinely compared with those expected for closely related taxa. Species-level identification might have been unsuccessful for several reasons, including lack of separation between closely related species (which resulted in a report of >1 species), poor sequence quality on multiple attempts, insertions or deletions in multiple nonidentical copies of the 16S rRNA gene (which compromised sequence quality, length, or both), unpublished or unsubstantiated references, or a lack of similar sequences in reference databases. After multiple isolates recovered from the same patients were eliminated, 1,678 (≈6%) isolates were found that had not been identified to the species level. A cutoff of <99% identity with a known species was used to define isolates that may represent novel taxa (*17**,**23*). On the basis of provided information, anatomical sites were classified as follows: blood, bones (including bone marrow), central nervous system (brain, cerebrospinal fluid), eye, gastrointestinal tract (abdomen, gallbladder, stool), genitourinary tract (genitals, placenta, urine), oral cavity/paranasal sinus (including throat), respiratory tract (invasive: bronchoalveolar lavage, bronchial brush/wash, lung; other: sputum, endotracheal aspirate, respiratory specimen), tissue, wound/abscess (including bite wounds, lesion, scraping), other (aspirate, biopsy, body and dialysis fluids, ear, heart valve, medical devices), or unknown.

### Sequence Assembly

Partial 16S rRNA gene sequencing had been performed as reported (*25*). Original chromatogram files were reanalyzed with MicroSeq 500 software (version 2.0; Applied Biosystems, Foster City, CA, USA). Consensus sequences of <400 bp in length were eliminated from further analyses. Remaining sequences with average phred quality scores >35 were included without manual review. Sequences with quality scores <35 were reviewed manually and included only if quality was sufficient, as determined by visual inspection. Sequences were converted to FASTA format (http://blast.ncbi.nlm.nih.gov/blastcgihelp.shtml) for comparison with reference sequences and submitted to GenBank under accession nos. JQ259197–JQ259857X and JN986812–JN986825. Sequences were annotated with taxonomic information from the best match with species-level identification by using CLSI guidelines (*14*). In brief, isolates with 97% to <99% identity were annotated at the genus level, isolates with 95% to <97% identity were annotated at the family level, and isolates with <95% identity were annotated at the order level. Aerobic actinomycetes (*26*), members of the family *Enterobacteriaceae*, and mycobacteria with identities of 95%–99% were annotated at the family level (*14*).

### Comparison to Reference Sequences

NCBI stand-alone-BLASTn version 2.2.23+ with default parameters and internally developed software applications were used to compare sequences to a local copy of the NCBI nucleotide database (*13*) (downloaded July 2010). Information from 3 matches per isolate was parsed from XML-formatted BLASTn output files into a database by using custom python code and biopython libraries (*27*): 1) top match with valid species-level annotation (e.g., *Streptococcus sanguinis*); 2) top match with valid genus-level annotation (e.g., *Streptococcus* sp. oral strain T4-E3); and 3) top BLASTn match irrespective of annotation (e.g., uncultured bacterium). Valid nomenclature was determined by comparing annotations in the GenBank organism field to a list of approved bacterial taxa (*28*). Values in the following GenBank database fields or BLAST XML results were retrieved from each of the 3 matches: organism, taxonomy, associated publication, publication date, alignment length, number of identities, and position in the hit list. Reference sequences with species-level annotation were used, whether they were linked to a publication or not. For each of the 3 matches, the number of ambiguous bases (International Union of Pure and Applied Chemistry codes) and the percent aligned (alignment length as percentage of query length) were calculated. Percent identity was calculated by considering International Union of Pure and Applied Chemistry ambiguity codes as matching any corresponding bases (e.g., Y matched C or T). N symbols were always recorded as mismatches.

Only sequences that had <99% identity with a valid species-level reference were included in subsequent analyses. Since BLASTn uses a local alignment algorithm, resulting alignments may be based on truncated query or match sequences if similarities are low at either end of the sequences. This practice may cause inflated pairwise sequence identity values. To control for this effect, we also retrieved the 3 matches described above using a minimum alignment length cutoff of 98%, on the basis of the query sequence length. Manual reviews were performed when this filter resulted in different best matches. For sequences with percent identity values close to the 99% cutoff and BLASTn alignment length of <100%, pairwise alignments with the best species-level match were analyzed by using MEGA4.1 (*29*). Percent identity was calculated manually for these isolates on the basis of a full-length alignment of query and match sequences.

### Phylogenetic Analysis to Determine Repeatedly Encountered Taxa

Isolates that likely belonged to the same undescribed species were recognized by constructing phylogenetic trees with related isolates in MEGA. Groups of isolates with high sequence identity were specified from phylogenetic trees, and percent identity was calculated from multiple sequence alignments by using MEGA. Isolates that shared >99.0% sequence identity with each other were considered part of the same cluster. For all clusters containing >5 isolates, BLASTn matches were manually reviewed. Phylogenetic trees were constructed by using sequences from clinical isolates in the same cluster and related type strains as identified by the The All-Species Living Tree Project (release 102) (*30*) and/or List of Prokaryotic Names with Standing in Nomenclature (*31*).

## Results

### Clinical Study Isolates

**Figure 1:**
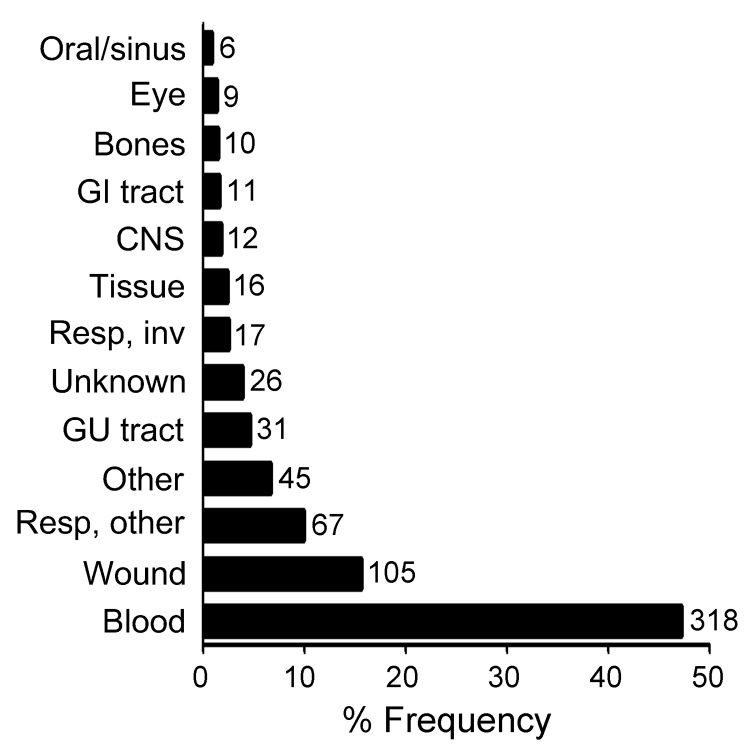
Anatomical sites that yielded 673 unidentified clinical bacterial isolates. The x-axis indicates relative frequency. Numbers to the right of bars represent isolate counts. GI, gastrointestinal; CNS, central nervous system; resp, respiratory; inv, invasive; GU, genitourinary.

During a 4-year period, 1,678 clinical isolates (≈6%) were not identified to the species level by routine 16S sequence analysis. Reanalysis of these sequences showed that 315 isolates (19%) were unidentified because of inadequate sequence quality; they were excluded from this study. The remaining 1,363 sequences were re-screened by using a current NCBI nucleotide database, and 690 (50.6%) were found to share >99% identity with >1 species-level annotated GenBank reference. The remaining 673 isolates were marked as probable novel taxa and included in this study. Of these 673 isolates, 52 (7.7%) were obtained at the University of Utah Medical Center, and the remaining isolates were referred from hospitals in 41 different US states. Nearly half of the isolates (47.3%) originated from blood cultures. Anatomical sources of the isolates are shown in Figure 1.

### Sequence Length and Quality

**Figure 2:**
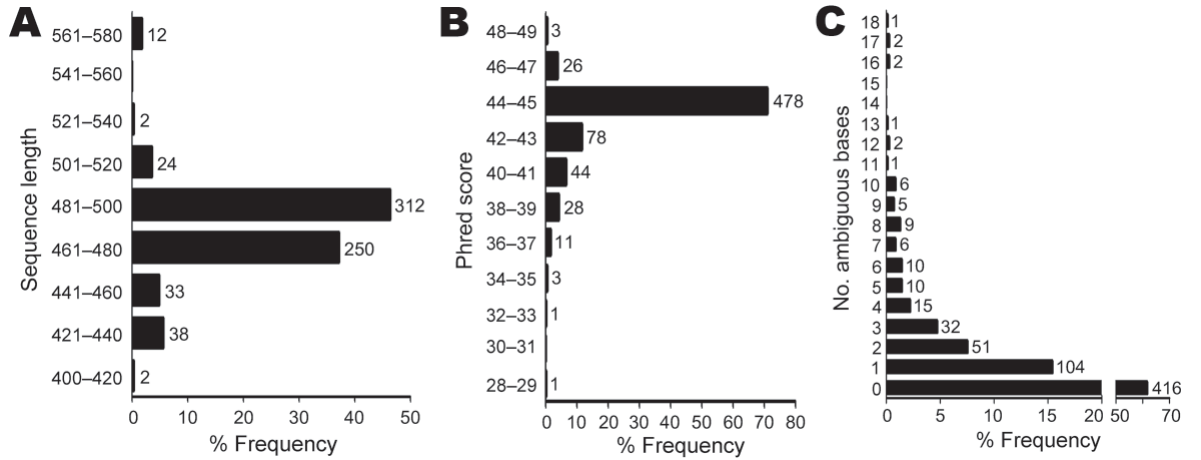
Sequence quality and number of ambiguous bases for 673 unidentified bacterial isolates. The median sequence length was 480 bases, with 84% of sequences in the range of 461 to 500 bases (A). The median phred sequence quality score was 45 (B). Most sequences had no ambiguous positions (n = 416, 61.8%). Up to 18 ambiguous positions were seen in isolates with multiple, nonidentical copies of the 16S rRNA gene (C). The x-axes indicate relative frequency. Numbers above columns represent isolate counts.

Most sequences (84%) for the 673 isolates had lengths of 460 to 500 bp, as expected on the basis of the PCR and sequencing primers used (Figure 2, panel A). The median sequence phred quality score for the isolates suspected of representing novel taxa was 45, indicating high-quality sequences (Figure 2, panel B). One to 18 ambiguous nucleotide positions were observed in 38% of isolates (Figure 2, panel C), indicating multiple nonidentical copies of the 16S rRNA gene.

### Similarity of Clinical Isolates to Reference Sequences

**Figure 3:**
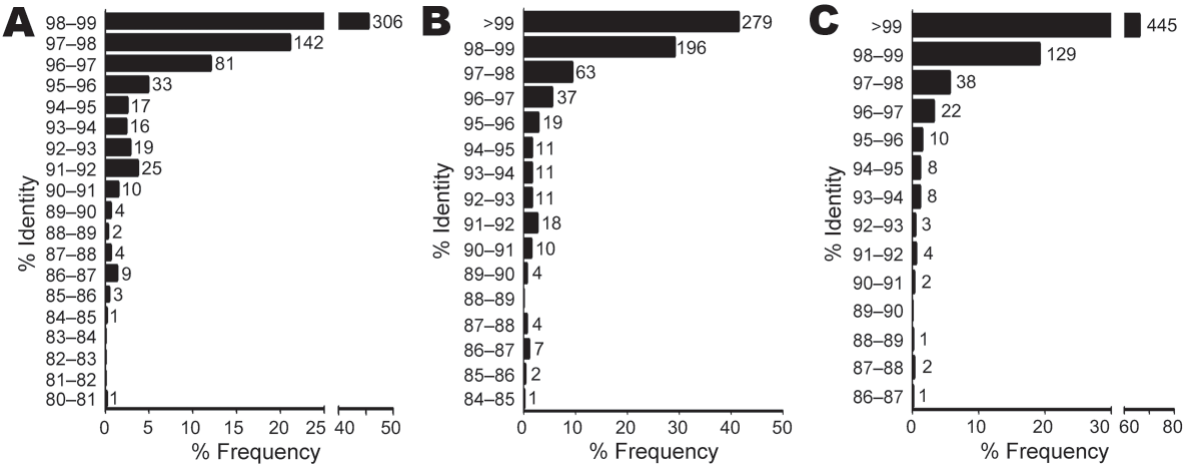
Identities of 673 unidentified bacterial isolates to best match in BLASTn database (*23*) with species-level (A) or genus-level annotation (B) and identity to best match in database, regardless of annotation status (C). The x-axes indicate relative frequency. Numbers to the right of bars represent isolate counts.

BLASTn identities were 80.9%–98.9% for references with valid species annotation (Figure 3, panel A), 84.5%–100% for references with valid genus annotation (Figure 3, panel B), and 86.7%–100% for any reference (Figure 3, panel C). A total of 448 isolates (66.6%) ranged from >97% to <99% identity to a valid species reference (*23*), likely indicating new species. However, fully one third of the isolates (n = 225) were <97% identical to a validly described species, satisfying a more conservative threshold for novel species (Figure 3, panel A) (*15*). Identities of 111 isolates (16.5%) were <95%, indicating novel genera (*21*). Using reference sequences with at least a genus-level annotation, we found that identities were >99% for 279 isolates (41.5%), >97% to <99% for 259 (38.5%), and <97% for 135 (20.1%) isolates (Figure 3, panel B). The same comparison with any reference, regardless of annotation, yielded values of 445 (66.1%), 165 (24.5%), and 61 (9.1%) isolates (Figure 3, panel C), with the latter group representing isolates highly divergent from any previously sequenced organisms.

### Taxonomic Analysis of Clinical Isolates Representing Novel Taxa

**Figure A1:**
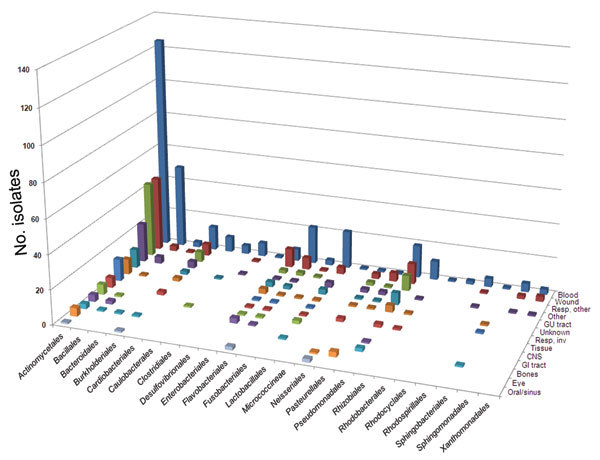
Order-level taxonomic information for 673 bacterial isolates is summarized on the basis of the anatomical source. Numbers represent isolate count**.**

Taxonomy of the 673 isolates was inferred from best database matches with species-level annotation (Table 1). The largest number of isolates (n = 294, 43.7%) belonged to the order *Actinomycetales*, followed by *Bacillales* (n = 61) and *Pseudomonadales* (n = 56). Within the order *Actinomycetales*, the most common families were *Actinomycetaceae* (n = 73), *Corynebacteriaceae* (n = 59), and *Nocardiaceae* (n = 53) (Table A1). Taxonomic information by source is summarized in Figure A1.

**Table 1:** Taxonomic distribution, by order of best species-level matches, for 673 isolates of possibly novel species of bacteria

Order	No. isolates
Actinomycetales	294
Bacillales	61
Pseudomonadales	56
Flavobacteriales	41
Burkholderiales	39
Lactobacillales	38
Enterobacteriales	33
Neisseriales	15
Pasteurellales	14
Rhizobiales	14
Clostridiales	10
Cardiobacteriales	9
Sphingomonadales	8
Caulobacterales	7
Rhodospirillales	7
Xanthomonadales	7
Fusobacteriales	6
Bacteroidales	5
Sphingobacteriales	4
Rhodocyclales	2
Desulfovibrionales	1
Micrococcineae	1
Rhodobacterales	1

### Taxonomic Analysis of Novel Taxa Represented by Multiple Clinical Isolates

Overall, 348 isolates (52%) belonged to 95 novel taxa represented by >1 isolate. Cluster sizes ranged from 2 to 15, and sequence identities to species-level references ranged from 86.5% to 98.9% (Table 2; Table A2). Clusters within the order *Flavobacteriales* showed the greatest divergence from known species, with only 92.9% average identity. Not surprisingly, given the preponderance of isolates in this order, the largest number of clusters (n = 45) was identified among the *Actinomycetales* (Table A1). Fourteen clusters with up to 9 members were seen in the family *Nocardiaceae*, 12 clusters with up to 12 members in *Actinomycetaceae*, and 9 clusters with up to 10 members in *Corynebacteriaceae*.

**Table 2:** Tentative novel taxa represented by >5 clinical isolates*†

Family	Identity, %	Initial cluster size	Reviewed cluster size	Gram stain morphology	Result
*Micrococcaceae*	98.5	15	0	GPR	*Rothia aeria*, short reference sequence
*Actinomycetaceae*	98.7	12	11	GPR	1 strain with >1% dissimilarity
*Thermoactinomycetaceae*	91.8	12	12	GPR	Belong to *Kroppenstedtia eburnea* gen. nov., sp. nov*.*
*Moraxellaceae*	96.4	11	11	GNR	Most similar to *Acinetobacter ursingii*
*Corynebacteriaceae*	98.1	10	10	GPR	Most similar to *Corynebacterium. mucifaciens*
*Corynebacteriaceae*	98.6	10	5	GPR	Most similar to *C. jeikeium,* 5 isolates are *C. jeikeium*
*Enterobacteriaceae*	98.9	10	10	GNR	Most similar to *Enterobacter cloacae*
*Streptomycetaceae*	98.5	9	0	GPR	*Streptomyces thermoviolaceus subsp. thermoviolaceus*
*Nocardiaceae*	98.9	9	9	GPR	Most similar to *Nocardia vermiculata*
*Cardiobacteriaceae*	98.9	8	0	GNR	Belong to *Cardiobacterium hominis*, poor reference sequence
*Flavobacteriaceae*	86.5	7	7	GNR	Most similar to *Chryseobacterium daecheongense*
*Actinomycetaceae*	96.9	7	7	GPR	Most similar to *Actinomyces odontolyticus*
*Actinomycetaceae*	98.5	6	6	GPR	Most similar to *Actinomyces meyeri*
*Thermoactinomycetaceae*	90.8	5	5	GPR	Most similar to *Laceyella putida*
*Actinomycetaceae*	95.0	5	3+2	GPR	2 separate taxa
*Streptococcaceae*	96.7	5	5	GPC	Most similar to *Streptococcus oralis*
*Enterobacteriaceae*	97.3	5	5	GNR	Most similar to *Dickeya dieffenbachiae*
*Actinomycetaceae*	97.8	5	3+2	GPR	2 separate taxa
*Streptococcaceae*	97.9	5	5	GPC	Most similar to *Streptococcus mitis*

*GPR, gram-positive rods; GNR, gram-negative rods; GPC, gram-positive cocci. †initial and reviewed clusters sizes indicate number of isolates in each cluster before and after manual review, outcome of manual review, and most similar valid species names are listed. Manual review was performed for all clusters with at least 5 isolates. Sequences were aligned with type strain sequences, and manual BLAST (*23*) analysis was performed to calculate pairwise sequence identities.

Nineteen novel taxa were represented by >5 isolates (Table 2). Upon manual review, 12 were confirmed without changes, 2 clusters contained at least 1 isolate with >1% sequence difference in pairwise comparisons, 2 clusters were split because of >1% sequence heterogeneity, and isolates of 3 clusters could be identified to validly described species: *Rothia aeria*, *Cardiobacterium hominis*, and *Streptomyces thermoviolaceus* subsp. *thermoviolaceus*. One cluster of 12 isolates belonged to a novel genus and species, *Kroppenstedtia eburnea*, which was described subsequent to our initial analysis (*32*).

### Anatomical Source of Unidentified Isolates

In addition to the frequency with which isolates of novel taxa are encountered in clinical specimens, their importance may also be judged by their anatomical source. Isolates cultured from the following normally sterile sites were considered clinically relevant: cerebrospinal fluid, pericardial fluid, synovial fluid, and tissues (brain, heart valve, or biopsy tissues). A total of 32 isolates were identified from these key sites. A manual analysis showed 3 isolates that were not identified because of short reference sequences and 1 isolate that was subsequently identified as *K. eburnea*. Of the remaining 28 isolates, 17 (61%) belonged to taxa that were repeatedly encountered. Taxonomic information for all 32 isolates is summarized in Table 3.

**Table 3:** Anatomical sites and possible novel bacterial isolates*

Source	Identity, %	Best species-level match	Cluster	Gram stain morphology	Comment†
Tissue	98.8	*Acidovorax delafieldii*	N	GNR	
Tissue	97.2	*Actinoallomurus fulvus*	N	GPR	
CSF	98.3	*Actinomyces meyeri*	Y	GPR	
Pericardial fluid	94.4	*Anaerococcus prevotii*	N	GPC	
Tissue	94.8	*Capnocytophaga sputigena*	N	GNR	
CSF	93.8	*Chryseobacterium taiwanense*	Y	GNR	*Planobacterium taklimakanense*, short reference sequence
CSF	98.3	*Corynebacterium mucifaciens*	Y	GPR	
Tissue	98.6	*Cupriavidus gilardii*	Y	GNR	
CSF	97.1	*Erwinia chrysanthemi*	Y‡	GNR	
Tissue	97.7	*E. chrysanthemi*	Y‡	GNR	
CSF	97.2	*Globicatella sanguinis*	Y	GPC	
Tissue	96.2	*Kocuria kristinae*	Y	GPC	
CSF	92.4	*Desmospora activa*	Y	GPR	*Kroppenstedtia eburnea*
Synovial fluid	91.2	*Laceyella sacchari*	N	GVR	
Tissue	96.0	*Microbacterium thalassium*	Y‡	GPR	
Tissue	95.9	*M. thalassium*	Y‡	GVR	
Tissue	96.9	*Neisseria canis*	N	GNC	
Tissue	97.9	*Neisseria zoodegmatis*	Y	GNCB	
Biopsy specimen	98.7	*Nocardia beijingensis*	Y	GPR	
Brain	98.9	*Nocardia nova*	Y	GPR	
Tissue	98.9	*Nocardia transvalensis*	N	GPR	
CSF	96.1	*Phenylobacterium immobile*	N	GNR	
Tissue	97.5	*Prosthecomicrobium enhydrum*	N	GVR	
Tissue	95.2	*Pseudomonas pohangensis*	Y‡	GNR	
Tissue	95.2	*Pseudomonas pohangensis*	Y‡	GNR	
CSF	98.5	*Rothia dentocariosa*	Y	GPR	*Rothia aeria*, short reference sequence
Tissue	98.0	*Streptococcus mitis*	Y	GPC	
Valve	96.8	*Streptococcus oralis*	Y	GPC	
CSF	98.0	*Streptococcus sanguinis*	Y	GPC	
CSF	96.4	*Streptomyces prasinopilosus*	N	GPR	
CSF	96.8	*Terrabacter terrae*	N	GPC	
Tissue	97.8	*Williamsia serinedens*	N	GPR	*Williamsia deligens*, short reference sequence

*GNR, gram-negative rods; GPR, gram-positive rods; CSF, cerebrospinal fluid; GPC, gram-positive cocci; GVR, gram-variable rods; GNC, gram-negative cocci; GNCB, gram-negative coccobacilli; Y, isolates belonging to tentative novel taxa represented multiple times in this study. †Results of manual review of BLASTn analysis (*23*). ‡These pairs of isolates belong to the same 3 respective clusters.

## Discussion

Broad-range molecular identification methods have facilitated the discovery of novel bacterial species and have resulted in a rapid increase in recognized bacterial taxa (*28*). The use of these methods in diagnostic laboratories may lead to the detection of bacterial strains that belong to novel species. We reviewed 16S sequencing results for >26,000 clinical isolates in a systematic approach to recognize novel species that may be pathogenic. Their formal description will provide the basis for improvements of sequence databases, antimicrobial susceptibility studies, and epidemiologic surveys to characterize their pathogenicity.

A sequence identity cutoff of <98.7%–99.0% for species discrimination has been shown to correlate with DNA-DNA hybridization results and is recommended for taxonomic purposes (*17**,**23*). In this study, 673 isolates showed <99% sequence identity and 535 isolates showed <98.7% sequence identity to any reference sequence with species-level annotation in the NCBI nucleotide database and could thus be considered novel taxa. Comparison of these sequences against the NCBI nucleotide database, the largest reference sequence repository (*10**,**11*), which contains 16S sequences for all newly described bacterial species, ensured a robust analysis of possibly novel species. Our algorithm employed 2 quality assurance criteria for reference sequences identified in BLASTn analysis: minimal alignment length of 98% and annotation as a validly described bacterial taxon (*28*). Because a more stringent manual review of reference sequences, as performed in diagnostic practice (*14*), was not feasible for this large study, the 673 isolates detected by this algorithm represent a conservative estimate of the total number of novel species encountered.

To ensure that sequence quality was not limiting, we confirmed that sequences were of expected length (Figure 2, panel A) and had phred scores showing a median accuracy of >99.99% per base (Figure 2, panel B). It has been recommended that sequences used for bacterial identification should contain <1% ambiguous positions (*19*), which was the case in 92% of the sequences in our study (Figure 2, panel C). However, ambiguous positions can be seen in bacteria with multiple, nonidentical 16S alleles. We observed up to 18 ambiguous positions in a small number of isolates (Figure 2, panel C), which is consistent with whole-genome sequencing data that indicate >19 nucleotide differences in bacteria with multiple rRNA operons (*33**,**34*). Although full-length 16S sequencing might have facilitated the identification of some isolates, partial 16S sequencing is considered robust (*10*) and is an unlikely reason for incomplete identification in most cases.

To determine taxonomic properties of all 673 isolates, we calculated 16S sequence identities to reference sequences with valid species-level (Figure 3, panel A), genus-level (Figure 3, panel B), or any annotation (Figure 3, panel C). Consistent with results of previous smaller studies, our results showed that most isolates were gram-positive rods and nonfermenting gram-negative rods (Table 1) (*22**,**35*). A total of 294 isolates belonged to the order *Actinomycetales*, with *Actinomyces* (n = 71), *Corynebacterium* (n = 59), and *Nocardia* (n = 52) being the most common genera. Molecular identification methods have resulted in a dramatic increase in the number of recognized species in these genera, and our results indicate that more species of possible clinical relevance are yet to be described (*28*). A total of 535 (79.5%) and 225 isolates (33.4%) belonged to novel species even when more conservative cutoffs of 98.7% and 97% identity, respectively, were used (*15**,**23*). Of these, 111 isolates (16.5%) represented novel genera at the conservative 95% identity cutoff (*10**,**21*).

To determine the isolates most likely to be of clinical importance, we identified novel taxa that were isolated repeatedly or were from normally sterile, clinically relevant anatomical sites. More than half of the unidentified organisms were isolated at least twice, forming clusters that represented 95 novel taxa. Most clusters belonged to the order *Actinomycetales* (45 clusters, 176 isolates), with 14 clusters (42 isolates) in the genus *Nocardia* and 12 clusters (52 isolates) in the genus *Actinomyces*. A total of 19 clusters that contained >5 members were initially identified (total of 156 isolates, Table 2). After manual review, isolates in 2 of these clusters were found to belong to validly described species (Table 2). These species were not identified in the automated analysis due to short reference sequences or because they had a subspecies annotation not covered in the algorithm. The validity of our approach was confirmed, however, when a novel thermoactinomycete, *Kroppenstedtia eburnea* (*32*), was formally described during preparation of this article. The 16S sequence of this organism showed ≈99.5% identity to a large cluster of 12 isolates in our study (Table 2).

While this study only included bacterial strains from clinical specimens (Figure 1), isolates from some anatomical sites (e.g., central nervous system) may be more likely to represent pathogens than others (e.g., upper respiratory tract). When highly stringent criteria are used (e.g., recovery from a normally sterile fluid or tissue), a minimum of 28 isolates may represent novel pathogens (Table 3). The presence of multiple isolates for 17 of these novel species further supports their status as potential pathogens. While proving pathogenicity is beyond the scope of this study, our analysis may serve as a sentinel for novel organisms with pathogenic potential and provide a rationale for further studies to define their pathogenicity.

During 2001–2007, a total of 215 novel bacterial species and 29 novel genera isolated from clinical samples were formally described (*1*). Only 100 of these new species were represented by at least 4 isolates, of which *Mycobacterium* and *Nocardia* were the most common genera. In contrast to our study, most new species were isolated from nonsterile body sites, such as the oral cavity and gastrointestinal tract, and may thus be commensal or from the environment. Using a proposed minimum of 3 to 5 isolates to describe novel bacterial species (*10**,**36**,**37*), the present study may include up to 46 novel species (<99% identity) and up to 4 novel genera (<95% identity). Alternatively, it has been argued that even a single isolate from a human specimen should be reported to allow for more rapid identification of additional isolates in other laboratories (*1**,**12**,**22*). By this strategy, several hundred novel taxa may be represented in this study. Although our study does not prove that these isolates represent novel species, it provides a framework for screening large numbers of sequences for possible novel taxa that may be of clinical importance. Candidate isolates will require rigorous polyphasic validation, including full 16S rRNA gene sequencing, to confirm that they are new bacterial species. By providing information on morphologic characteristics, antimicrobial drug susceptibility profiles, virulence factors, and spectrum of disease, future studies will facilitate clinical decision making. Results of our phylogenetic analysis may thus help focus efforts to formally describe novel, clinically relevant species and to improve the diagnostic utility of reference databases.

## Appendix

**Table A1:** Tentative novel species of bacteria represented by multiple isolates (clusters) for the different families in the order *Actinomycetales*

Taxon (family)	In clusters							Not in clusters			Total	
	No. (%) isolates	Identity, %*	No. clusters	Cluster size				No. isolates	Identity, %*		No. isolates	Identity, %
Min	Max	Med
*Actinomycetaceae*	52 (71)	97.9	12	2	12	3.5		21	96.5		73	97.5
*Nocardiaceae*	42 (79)	98.6	14	2	9	2		11	98.7		53	98.6
*Corynebacteriaceae*	40 (68)	98.1	9	2	10	3		19	97.6		59	97.9
*Micrococcaceae*	17 (81)	98.2	2	2	15			4	97.8		21	98.2
*Microbacteriaceae*	14 (45)	97.3	6	2	3	2		17	96.5		31	96.9
*Streptomycetaceae*	9 (45)	98.5	1	9				11	97.6		20	98.0
*Pseudonocardiaceae*	2 (67)	95.9	1	2				1	97.2		3	96.4
*Intrasporangiaceae*								6	97.8		6	97.8
*Cellulomonadaceae*								5	96.9		5	96.9
*Thermomonosporaceae*								4	97.4		4	97.4
*Dermabacteraceae*								3	96.3		3	96.3
*Mycobacteriaceae*								3	97.7		3	97.7
*Propionibacteriaceae*								3	92.5		3	92.5
*Gordoniaceae*								2	98.5		2	98.5
*Nocardioidaceae*								2	97.2		2	97.2
*Williamsiaceae*								2	97.8		2	97.8
*Brevibacteriaceae*								1	98.9		1	98.9
*Dermacoccaceae*								1	93.7		1	93.7
*Sanguibacteraceae*								1	97.9		1	97.9
*Tsukamurellaceae*								1	96.8		1	96.8
Total	176 (60)	98.1						118	97.1		294	97.7

*Sequence identity to the closest GenBank match with species designation (match %). Min, minimum; max, maximum; med, median.

**Table A2:** Tentative novel species of bacteria represented by multiple isolates (clusters)

Taxon	In clusters							Not in clusters			Total	
	No. (%) isolates	Identity, %*	No. clusters	Size				No. isolates	Identity, %*		No. isolates	Identity, %
				Min	Max	Med						
*Actinomycetales*	176 (60)	98.1	45	2	15	3		118	97.1		294	97.7
*Pseudomonadales*	30 (54)	97.1	8	2	11	2.5		26	96.8		56	97.0
*Lactobacillales*	28 (74)	97.9	8	2	5	3.5		10	95.1		38	97.1
*Bacillales*	27 (44)	96.5	7	2	12	2		34	94.1		61	95.1
*Flavobacteriales*	20 (49)	92.9	6	2	7	3		21	95.6		41	94.3
*Enterobacteriales*	19 (58)	98.3	4	2	10	3.5		14	98.0		33	98.2
*Burkholderiales*	15 (38)	97.3	6	2	4	2		24	97.6		39	97.4
*Cardiobacteriales*	8 (89)	98.9	1	8				1	97.5		9	98.7
*Rhizobiales*	6 (43)	93.5	2	2	4			8	97.0		14	95.5
*Neisseriales*	5 (33)	97.9	2	2	3			10	96.9		15	97.3
*Fusobacteriales*	3 (50)	98.7	1	3				3	93.5		6	96.1
*Rhodospirillales*	3 (43)	97.2	1	3				4	96.9		7	97.0
*Clostridiales*	2 (20)	97.7	1	2				8	94.3		10	95.0
*Pasteurellales*	2 (14)	98.9	1	2				12	96.2		14	96.6
*Sphingomonadales*	2 (25)	97.9	1	2				6	97.1		8	97.3
*Xanthomonadales*	2 (25)	95.5	1	2				5	97.1		7	96.7
*Caulobacterales*								7	97.6		7	97.6
*Bacteroidales*								5	92.3		5	92.3
*Sphingobacteriales*								4	87.0		4	87.0
*Rhodocyclales*								2	95.5		2	95.5
*Desulfovibrionales*								1	94.6		1	94.6
*Micrococcineae*								1	97.4		1	97.4
*Rhodobacterales*								1	98.4		1	98.4
Total	348 (52)							325			673	

*Sequence identity to the closest GenBank match with species designation (match %). Min, minimum; max, maximum; med, median.
